# Hair Mercury Levels in U.S. Children and Women of Childbearing Age: Reference Range Data from NHANES 1999–2000

**DOI:** 10.1289/ehp.7046

**Published:** 2004-05-27

**Authors:** Margaret A. McDowell, Charles F. Dillon, John Osterloh, P. Michael Bolger, Edo Pellizzari, Reshan Fernando, Ruben Montes de Oca, Susan E. Schober, Thomas Sinks, Robert L. Jones, Kathryn R. Mahaffey

**Affiliations:** ^1^National Center for Health Statistics, Centers for Disease Control and Prevention, Hyattsville, MD, USA; ^2^National Center for Environmental Health, Centers for Disease Control and Prevention, Atlanta, Georgia, USA; ^3^Center for Food Safety and Applied Nutrition, U.S. Food and Drug Administration, College Park, Maryland, USA; ^4^Research Triangle Institute, Research Triangle Park, NC, USA; ^5^The Orkand Corporation, Falls Church, Virginia, USA; ^6^The Office of Science Coordination and Policy, Office of Prevention, Pesticides, and Toxic Substances, U.S. Environmental Protection Agency, Washington, DC, USA

**Keywords:** blood, child, diet, female, hair, mercury, NHANES, preschool

## Abstract

Exposure to methyl mercury, a risk factor for neurodevelopmental toxicity, was assessed in U.S. children 1–5 years of age (*n* = 838) and women 16–49 years of age (*n* = 1,726) using hair mercury analysis during the 1999–2000 National Health and Nutrition Examination Survey (NHANES). The data are nationally representative and are based on analysis of cross-sectional data for the non-institutionalized, U.S. household population. The survey consisted of interviews conducted in participants’ homes and standardized health examinations conducted in mobile examination centers. Distributions of total hair mercury levels expressed as micrograms per gram hair Hg and the association of hair Hg levels with sociodemographic characteristics and fish consumption are reported. Geometric mean (standard error of the geometric mean) hair mercury was 0.12 μg/g (0.01 μg/g) in children, and 0.20 μg/g (0.02 μg/g) in women. Among frequent fish consumers, geometric mean hair mercury levels were 3-fold higher for women (0.38 vs. 0.11 μg/g) and 2-fold higher for children (0.16 vs. 0.08 μg/g) compared with nonconsumers. The NHANES 1999–2000 data provide population-based data on hair mercury concentrations for women and children in the United States. Hair mercury levels were associated with age and fish consumption frequency.

Mercury is a naturally occurring heavy metal whose presence in the environment is widespread and persistent [Agency for Toxic Substances and Disease Registry (ATSDR) 1999; National Research Council (NRC) 2000]. Hg occurs in metallic or elemental, inorganic, and organic forms (ATSDR 1999). When elemental Hg is emitted as a combustion by-product of fossil fuels, it becomes methylated in the environment and accumulates in animal tissues, including fish. Methyl mercury (MeHg) in the aquatic food chain contributes to higher tissue Hg levels among fish consumers (Boening 2000). Total Hg in the hair of fish eaters correlates with Hg in the target tissue, the brain (Cernichiari et al. 1995).

The mammalian nervous system is highly vulnerable to MeHg (Castoldi et al. 2001). Exposure to high levels of MeHg during the last two trimesters of pregnancy produces documented neurodevelopmental problems, including language, attention, and memory problems [Marsh et al. 1987; NRC 2000; U.S. Environmental Protection Agency (EPA) 1997]. Accidental poisoning incidents in Japan (Harada 1995) and Iraq (Amin-Zaki et al. 1974) demonstrated the pronounced neurologic injuries that result from high-level MeHg exposures, particularly in children who were exposed *in utero*.

More recent prospective epidemiologic data from New Zealand, the Faroe Islands, and the Seychelles assessed developmental effects of lower level MeHg exposure in fish-consuming populations resulting from maternal and fetal exposures to MeHg (Cernichiari et al. 1995; Grandjean et al. 1999). The U.S. EPA MeHg exposure reference dose (RfD) of 0.1 μg/kg body weight/day was based on data from the Faroes and New Zealand, with supporting analyses from all three major prospective cohort studies (Rice et al. 2003). The assessments of Hg exposure for the U.S. population have included regional biomonitoring studies conducted by state and federal agencies (Pellizzari et al. 1999) and assessments of population subgroups, including sport fisher-men and their families (Burge and Evans 1994; Kosatsky et al. 2000), pregnant women in selected geographic areas (Björnberg et al. 2003; Stern et al. 2001), high-end fish consumers (Hightower and Moore 2003), and American Indian and Alaskan Native groups (Rothschild and Duffy 2001).

Total blood and hair Hg are indicators of MeHg exposure in fish consumers and others who are not exposed to inorganic and elemental Hg occupationally or incidentally (Carrington and Bolger 2002; Mahaffey 2000; NRC 2000). Exposure to MeHg increases with fish consumption [International Programme on Chemical Safety (IPCS) 1990; Yamaguchi et al. 1975]. Once consumed, 90% of MeHg is absorbed in the human gut (Miettinen 1973). Approximately 95% of measurable Hg in blood is the methylated form (Sherlock et al. 1984). After absorption, MeHg is distributed throughout the body within hours (Clarkson 1997). Peak MeHg blood levels in human subjects fed fish containing known concentrations of MeHg occurred within 4–14 hr of ingestion (Kershaw et al. 1980). The mean ± SE half-life of blood MeHg reported from a study with 20 adults whose diet included halibut with measured MeHg was 50 ± 1 days (range, 42–70 days) (Sherlock et al. 1984).

Hair Hg concentration is the preferred biomarker for evaluating Hg exposure for extended periods of time such as periods of weeks or months (NRC 2000). Hair incorporates Hg present in circulating blood during hair formation in the hair follicle (Clarkson 1983). Hair growth and analysis studies assessed hair growth rates and the relationship between MeHg intake and hair levels (Clarkson 1992; Grandjean et al. 1994). Hair growth averages 1–1.5 cm per month and provides a time record of previous Hg exposure depending on the length of the hair (Suzuki et al. 1984). Approximately 80% of hair Hg is MeHg (Cernichiari et al. 1995; Phelps et al. 1980). Total Hg and MeHg levels in hair are linearly related (Pellizzari et al. 1999), with total Hg concentrations in hair thought to average 150- to 200-fold higher than Hg concentrations in blood (Gill et al. 2002).

The Centers for Disease Control and Prevention (CDC), the U.S. EPA, U.S. Food and Drug Administration, Department of Energy, and the National Oceanographic and Atmospheric Administration funded a comprehensive Hg assessment component as part of the 1999–2001 National Health and Nutrition Examination Survey (NHANES). The NHANES Hg assessment component targeted children 1–5 years of age and women 16–49 years of age. Preliminary blood and hair Hg results from NHANES 1999 were reported in 2001 (CDC 2001). A detailed analysis of NHANES 1999–2000 blood Hg data was published in 2003 (Schober et al. 2003). Blood Hg levels were examined by race/ethnicity, maternal education level, and fish consumption frequency. Blood Hg levels were low overall in children and adults, but approximately 8% of women had levels > 5.8 μg/L, a level that corresponds to the U.S. EPA’s RfD. At the time, geometric mean (GM) total hair Hg values were not computed for the NHANES 1999 data. This report on hair Hg describes hair Hg levels in U.S. children and women of childbearing age by race/ethnicity, fish consumption frequency level, pregnancy status, and education level (females 20–49 years of age) and examines the relationship between total blood and hair Hg.

## Materials and Methods

### Survey description.

The NHANES survey is conducted to provide continuous health and nutrition data on the U.S. population for all ages [National Center for Health Statistics (NCHS) 2002b]. Standardized interviews and examination methods are administered by trained staff, including physicians, dentists, health technologists, interviewers, and laboratory technicians. Health examinations are conducted in mobile examination centers that travel to 15 geographic sites per year.

### Sample design.

The NHANES survey design is a stratified, multistage, national probability sample of the civilian, noninstitutionalized U.S. population; regional estimates are not produced (NCHS 2002). One or more participants within the eligible households sampled are included in the survey. Annual samples are nationally representative and include approximately 6,000 interviewed and 5,000 examined persons. NHANES 1999–2001 included expanded samples of Mexican Americans, non-Hispanic blacks, pregnant females, adolescents 12–19 years of age, and adults ≥ 60 years of age.

### Data collection for the Hg assessment component.

The NHANES 1999–2000 Hg assessment component included a dietary interview and blood, hair, and urinary Hg assessments. Venipuncture blood collection was conducted on persons ≥ 1 year of age, hair collection on children 1–5 years of age and women 16–49 years of age, and urine collection on persons ≥ 6 years of age. The NHANES dietary assessment component included a 24-hr dietary recall interview on all examined persons and fish and shellfish consumption frequency questions on persons ≥ 1 year of age. All survey examination protocols, questionnaires, and reporting criteria were approved by the NCHS institutional review board; total hair Hg values of ≥ 15 μg/g or total blood Hg values of ≥200 μg/L were reported to participants. Signed informed consent was obtained for all survey participants. Participants or their guardians provided consent for participants < 18 years of age. Examined persons received remuneration for their participation in the survey ranging from $30 to $100, depending on their age and examination content; transportation and child care expenses were also compensated.

### Hair specimen collection.

A minimum of 5–10 mg of hair was required for the hair analysis assay. Approximately 100 strands of hair (~ 50 mg) were gathered and cut from the occipital region of the scalp (approximately the diameter of a pencil eraser). A 1.5 × 2 in. Post-it (adhesive paper square; 3M, St. Paul, MN) was placed over the end of the hair strands closest to the scalp; the paper was marked with an arrow indicating the end of hair closest to the scalp. A plastic clip was placed over the paper to secure the hair sample. The samples were placed in a resealable plastic bag and shipped to the Research Triangle Institute for analysis. Respondents or proxies for children were asked if the respondent’s hair had been given a permanent or been treated with a hair dye or a hair relaxer product within the last month. The analysis compared total hair Hg in persons with treated hair (any of the products listed) to those with untreated hair.

### Hair Hg analysis.

Hair segments of 0.4 in. (1.0 cm) closest to the scalp, approximately 1 month’s growth, were analyzed for total Hg concentration using a cold vapor atomic fluorescence spectroscopy method (Pellizzari et al. 1999). The method involves digestion of the analyte from hair samples using a 30:70 mixture of sulfuric and nitric acids and subsequent analysis by cold vapor atomic fluorescence spectrometry. The analyte is identified by the presence of fluorescence signal from a Hg-specific detector. During NHANES 1999–2000, hair Hg was analyzed in batches of 20–40 samples, and quantification of the analyte was carried out using batch-specific standard calibration curves. Linearity greater than 0.99 was confirmed for each curve using eight aqueous calibration standards (0, 5, 10, 30, 50, 80, and 85 pg/mL Hg) as previously described (Pellizzari et al. 1999). Daily quality control (QC) procedures included analysis in triplicate of a known human hair reference standard certified at 4.42 μg/g Hg (Pellizzari et al. 1999). QC standard checks were performed initially and after every 10th sample, and replicate measurements were performed (duplicate sample preparation with duplicate analysis of each preparation).

Percent recovery of the Hg analyte was monitored by analyzing hair samples spiked with a known Hg reference standard before the digestion process. Mean percent recovery of Hg (± SD) in the spiked samples was 96.2 ± 12.1%]. The precision of analysis was assessed from duplicate extracts (i.e., reanalysis of the same extract at a later time) and from duplicate hair sample analyses (i.e., a second aliquot of hair processed through the entire analysis process). The mean precision (± SD) for duplicate extract and sample analyses was 5.4 ± 8.7% and 11.7 ± 14.4%), respectively. The limit of detection (LOD) for total hair Hg varied by analytic batch because of the laboratory’s batch-specific standardization methodology. Method detection limits ranged from 0.0006 to 0.06 μg/g. Whenever the values for hair Hg were below a batch LOD, a fill value equal to the batch-specific LOD divided by the square root of 2 was used (Taylor 1987).

### Fish and shellfish consumption frequency questionnaire.

Fish and shellfish consumption frequency during the 30-day period before hair collection was reported. The fish and shellfish frequency questions were administered by bilingual interviewers in English or Spanish. A proxy respondent reported for children < 6 years of age and proxy-assisted interviews were conducted with children 6–11 years of age (NCHS 2002a). No information was obtained about portion sizes, recipes, or preparation methods. Fish frequency data were grouped into three categories: no fish consumed, fish consumed one or two times, and fish consumed three or more times during the past 30 days. Shellfish consumption was coded as either no shellfish consumed or shellfish consumed one or more times during the past 30 days. Weighted GM total hair Hg levels and percentiles were computed for each fish and shellfish consumption frequency category.

### Covariates.

Race and ethnicity were categorized as non-Hispanic white, non-Hispanic black, and Mexican American based on self-reported information provided by the survey participants. The sample size for the other race/ethnicity group that includes Asians and American Indians was too small to present separate estimates; the total population estimates include data for the other race/ethnicity group composed of 103 children and 195 women. Age in years at the time of the household interview was used. Pregnancy status was self-reported during private interviews administered to women ≥ 12 years of age at the mobile examination center. Pregnancy status was confirmed with a urinary pregnancy test in females ≥ 18 years of age in NHANES 1999 and in females ≥ 8 years of age in NHANES 2000.

### Statistical methods.

Weighted estimates were produced using NHANES mobile examination center–examined sample weight values. These NHANES 1999–2000 sample weights adjust for the differential probabilities of selection and nonresponse in the survey sample (NCHS 2002b). The sample weights were poststratified to the 1990 U.S. Census total population estimates. A second non-response adjustment was applied during the hair Hg analysis to adjust for the higher, non-random hair collection nonresponse rates among African-American and Mexican-American boys (Lohr 1999).

Distributions, extreme hair Hg values, influential observations based on an analysis of the survey sample weights and hair Hg data, laboratory QC data, and correlation analyses were examined initially (Korn and Graubard 1999). After the initial analyses demonstrated that the total hair Hg data were non-normally distributed, a logarithmic transformation of total hair Hg was applied to normalize the distribution data. Outliers and influential points were detected using box plots, normal probability plots, and residual analysis, including studentized residuals for hair Hg and log of hair Hg, weighted and unweighted. Percentiles, means, and GMs were calculated to describe the distributions of hair Hg levels in children and women; arithmetic and GMs were included for comparison with other published reports. Standard errors (SEs) of the GMs and means and their confidence intervals (CIs) were computed using SUDAAN (Survey Data Analysis) weighted delete-1 jackknife (Research Triangle Institute 2001). Tests of statistical significance used = 0.05. Weighted Pearson partial correlations were used for bivariate comparisons and to identify predictors for the multiple regression models. Correlations of total log blood and log hair Hg were performed using Pearson’s method. Total hair to total blood Hg ratios were computed for children and women. The natural logarithm of hair Hg was the dependent variable for all of the regression analyses. Multiple regression was performed using SUDAAN. Tests of differences among groups used regressions with groups defined by dummy variables described in multivariate analysis, and detection of predictors through regression (Srivastava and Sen 1990).

## Results

### Response rates.

A total of 12,160 persons were selected to participate in NHANES 1999–2000; 9,282 completed the household interview and health examination components (76.3%). Of the 1,250 children 1–5 years of age selected for the survey, 1,013 (81%) were interviewed and examined. Hair specimens were obtained from 838 examined children (83% of examined children); hair collection response rates were lower among non-Hispanic black and Mexican-American males because of insufficient hair specimen collection. Hair samples were obtained from 44 and 82% of non-Hispanic black and Mexican-American males respectively, compared with 90% of non-Hispanic white males. This response was not random, and sample weight adjustments were applied during final data analysis. A total of 2,314 women 16–49 years of age were selected to participate; of these, 1,819 were interviewed and examined (79%). Hair specimens were obtained from 1,726 women (95% of the examined women). Fish and shellfish consumption data were obtained for 93% of children (*n* = 785) and 96% of women (*n* = 1,660) with hair Hg data. Detectable total hair Hg levels were measured in 84 and 89% of hair specimens obtained for children and women, respectively.

### Outliers and influential points.

Three data points were considered outliers and influential. The values for hair Hg were 109.8, 415.2, and 849.0 μg/g for three Mexican-American participants who were 3, 1, and 37 years of age, respectively. Repeat hair Hg analyses confirmed the high hair Hg values, and laboratory QC specimens for the batches in which the specimens were analyzed were within specification. Blood Hg data were examined for the hair Hg outlier subset; total and inorganic blood Hg levels for all three persons were elevated, suggesting significant exposure to organic and inorganic Hg sources. Appropriate follow-up notification measures were undertaken to inform survey participants of these findings. Multivariate analyses with the outlier data did not alter the results appreciably. GMs, means, and medians increased slightly when the outlier values were included. For the purpose of reporting national reference values for total hair Hg, the hair Hg outlier values were excluded from further analysis.

### GMs and percentiles.

GMs, means, and percentiles of hair Hg levels for children and women, respectively, are presented in Tables 1 and 2. For children, data are presented by race/ethnicity, fish consumption, shellfish consumption, and hair treatment group (Table 1). For women, results are presented by race/ethnicity, age, fish consumption, shellfish consumption, hair treatment, pregnancy status, and education group (Table 2).

#### Race/ethnicity.

Among children, the overall GM total hair Hg was 0.12 μg/g. Non-Hispanic black and Mexican-American children had higher hair Hg levels than non-Hispanic white children. Boys had higher (non-significant) hair Hg levels than girls in all race/ethnicity groups (not shown). For women, non-Hispanic white females had significantly higher GM hair Hg levels than did non-Hispanic blacks and Mexican Americans.

#### Hair treatment.

The GM hair Hg level of children who received hair treatments during the past month did not differ from that of the untreated group. Thirty-seven percent of women reported using a hair treatment; the GMs total hair Hg levels of the treated and untreated hair groups were the same (0.20 μg/g).

#### Fish consumption.

GM hair Hg increased with increasing frequency of fish consumption for children and adults (Tables 1 and 2). The GM hair Hg level of children consuming fish three or more times during the past month was twice as high as for nonconsuming children (0.16 vs. 0.08 μg/g). Frequent fish consumers 30–49 years of age had the highest GM hair Hg levels of the adult groups examined (0.41 μg/g).

#### Pregnancy status.

GM hair Hg levels of pregnant women (*n* = 292) did not differ from that in nonpregnant women. Analysis by race/ethnicity among the pregnant sub-sample was limited to comparisons of GM and median hair Hg values because of small sample sizes; race/ethnicity differences were not statistically significant among the pregnant women. Non-Hispanic white and Mexican-American women had higher hair Hg levels than did non-Hispanic black women. GM hair Hg levels among the pregnant, frequent fish consumers (*n* = 79) did not differ from that among nonpregnant frequent fish consumers (0.56 vs. 0.37 μg/g, respectively).

#### Regression analysis.

Separate multiple linear regression models were developed for children and women (Tables 3 and 4). The reference group for the regression models was the non-Hispanic white group. Log-transformed hair Hg was regressed on race/ethnicity, fish consumption, shellfish consumption, and hair treatment for children (Table 3). Among children, differences were observed by race/ethnicity and fish consumption status. Compared with non-Hispanic whites, non-Hispanic blacks and Mexican Americans had higher total hair Hg levels. The least-squares (LS) means and their SEs were computed using the regression model *y* = log (hair Hg). LS means for non-Hispanic blacks and Mexican Americans were 0.19 and 0.16 μg/g, respectively, compared with 0.09 μg/g for non-Hispanic whites. Children who consumed fish one or more times during the previous month had higher total hair Hg levels than did children who did not consume fish. LS means for children (Table 3) who consumed fish one to two and three or more times per month were the same (0.14 μg/g) and can be compared with those of children who did not consume fish during the previous month (0.08 μg/g).

A separate regression model was developed for women 16–49 years of age (Table 4). Pregnancy status, age group, race/ethnicity, fish consumption, shellfish consumption, and hair treatment were included in the model. Hair Hg levels differed by race/ethnicity, and fish consumption and shellfish consumption frequencies. Log hair Hg levels of non-Hispanic whites and Mexican Americans were similar. Non-Hispanic whites had higher hair Hg levels than did non-Hispanic blacks. There was a positive relationship between fish consumption frequency and log hair Hg; log hair Hg levels of persons who consumed fish three or more times had higher total hair Hg levels than did women who consumed zero or one to two servings of fish during the past 30 days. When age groups were compared, hair Hg levels among women 40–49 years of age were higher than the reference group (women 16–19 years of age); the 20- to 29-and 30- to 39-year-old groups were not different from the reference group. LS means and 95% CIs were computed using the multiple regression model (Table 4). A separate analysis (not shown) was completed on a sub-sample of women 20–49 years of age to test the effects of education level as a surrogate marker for socioeconomic status. Education level was not associated with total hair Hg levels and did not alter the relationship between hair Hg and the other covariates.

### Hair-to-blood correlations and ratios.

Weighted Pearson correlations between log blood and log hair Hg were 0.67 for children and 0.79 for women, respectively (both correlations were *p* < .0001). Hair-to-blood Hg ratios were computed using total hair Hg (ng/g hair) and blood Hg (μg/L) data. The SUDAAN Proc Ratio procedure for correlated variables was used to compute the ratios (Research Triangle Institute 2001). Weighted delete-1 jackknife was used; the numerator and denominators were weighted sums for nonmissing values. The total sample mean ± SE hair-to-blood Hg ratios were 342 ± 20 for children 1–5 years of age and 234 ± 15 for females 16–49 years of age.

## Discussion

The NHANES 1999–2000 total hair Hg data provide national hair Hg reference data for U.S. children 1–5 years and women 16–49 years of age, including three major race/ethnicity subgroups. These baseline data will be used to monitor hair Hg levels in the U.S. population over time. These results were compared with reports across studies that used different biologic matrices to estimate Hg exposure.

The total hair Hg levels of NHANES children and women were generally lower than the levels reported in other studies of U.S. and international populations. GM hair Hg level in fish-consuming children 7 years of age in the Faroes was 2.99 μg/g (Grandjean et al. 1999) compared with a GM (SE) value of 0.16 (0.02) μg/g among frequent fish consumers in the NHANES sample 1–5 years of age. The mean ± SD and median maternal hair Hg levels of women in the Seychelles where frequent fish consumption occurs were 6.85 ± 4.5 μg/g and 5.94 μg/g, respectively (Cernichiari et al. 1995). The median total hair Hg level of women in the Faroes birth cohort study was 4.5 μg/g; 12% had levels > 10 μg/g (Grandjean et al. 1992). Recent hair Hg data for Japanese adult females residing in five districts showed an overall total hair Hg GM of 1.43 μg/g (range, 1.23–2.50 μg/g; Yasutake et al. 2003). The GM (SE) and median values for frequent fish consumers among women of childbearing age in NHANES were 0.77 (0.09) μg/g and 0.33 μg/g, respectively. The arithmetic mean hair Hg level reported in a probability-based sample of U.S. Great Lakes region residents ≥ 21 years of age was 0.29 μg/g (Pellizzari et al. 1999), compared with an arithmetic mean ± SE hair Hg value of 0.47 ± 0.06 μg/g for NHANES females 16–49 years of age.

We report that the GM total hair Hg among pregnant women was 0.21 μg/g and did not differ from hair Hg levels of nonpregnant women (GM = 0.20 μg/g). The mean ± SE hair Hg level reported in a study of 189 New Jersey pregnant women was 0.53 ± 0.07 μg/g (range, < 0.2–9.1 μg/g; Stern et al. 2001); the NHANES pregnant females had an arithmetic mean ± SE hair Hg of 0.43 ± 0.089 μg/g. Median hair Hg levels for 127 pregnant Swedish women were 0.35 mg/kg (range, 0.07–1.5 mg/kg; Björnberg et al. 2003). Prenatal assessments of women from the Seychelles women reported mean ± SD total hair Hg of 6.85 ± 4.5 ppm and a median value of 5.94 ppm (Myers et al. 2003).

These hair Hg values provide an estimate of exposure over an approximate 1 month period, as recent exposure is not yet incorporated into the hair growth outside of the scalp. The steady-state hair-to-blood MeHg concentration ratio reported by the World Health Organization for adults was approximately 250:1, compared with a total hair-to-blood Hg ratio of 234 for NHANES females (IPCS 1990). Values for NHANES children were higher (ratio value of 342 overall), and this may reflect the higher percentages of children with blood Hg levels below the LOD. The hair-to-blood ratios were highly variable, in part, because hair and blood measurements are not comparable regarding the time period of exposure. Additionally, Hg exposure in this study is low for most respondents, and these results may not be comparable with correlations and ratios predicted in groups with higher levels of Hg exposure.

The advantages of hair Hg assessment include the fact that hair collection is noninvasive, and good response rates can be achieved in population subgroups that are difficult to obtain blood specimens from, such as children. For example, during NHANES 1999–2000, blood Hg data were collected on 56% of selected children, whereas hair collection was completed on 67% of selected children. Additionally, hair is a time record marker of MeHg exposure in individuals and can be used to estimate Hg exposure over extended periods of time such as fetal exposure during gestation (Cernichiari et al. 1995).

Several considerations for interpreting the NHANES 1999–2000 hair Hg results are provided. The NHANES 1999–2000 sample, although nationally representative, does not permit estimation of MeHg exposure in population groups with potentially high dietary exposure such as subsistence fishers, residents in specific geographic regions of the United States, sport fishers, and members of racial and ethnic population subgroups (e.g., Asians and Pacific Islanders). These subgroups may consume more fish than the general U.S. population and have higher MeHg exposure. Seafood consumption among Asian Americans and Pacific Islanders (AAPI) in King County, Washington, averaged 117 g/day (Sechena et al. 2003), compared with mean intakes of 10–14 g/day for the total U.S. population ≥ 20 years of age (U.S. Department of Agriculture 1999). Significant variation was observed in consumption rates and food preferences of the 10 AAPI groups. Second, three extreme hair Hg values were analyzed and confirmed in NHANES and were discarded in developing the distributions because it was not possible to determine the contributing factors that resulted in these values. This underscores the complexity of Hg assessment and exposure in populations. Finally, the NHANES 1999–2000 sample design was composed of a small number of primary sampling units (PSUs; 26 unique PSUs total). This feature limits regional or geographic area comparisons and statistical comparisons between population subgroups.

The NHANES data may be useful for assessing the prevalence of health risks in the U.S. population when the associated risks of low-level Hg content are better defined and may be used to support diet and health research, policy, and monitoring activities. Diet remains the primary contributor to MeHg exposure in populations. More than 50% of NHANES participants consumed fish during a 30-day reference period. Annual seafood consumption projections for the U.S. population indicate that 75–93% of adult women and 58–72% of children 2–5 years of age consume seafood (Carrington and Bolger 2002).

Hair Hg analysis in national samples of U.S. children and women of childbearing age provide a useful biomarker for long-term Hg exposure. Acceptance of the hair collection procedure was high among survey participants and excellent method precision was achieved, allowing for of detection of hair Hg in approximately 84 and 89% of children and women, respectively. Total hair Hg is associated with age, race/ethnicity, and fish consumption frequency. Among women of childbearing age, total hair Hg levels of pregnant and non-pregnant women were the same.

## Figures and Tables

**Table 1:** GM and selected percentile (95% CI) total hair Hg (μg/g): U.S. children 1–5 years of age, NHANES 1999–2000.

				Percentile					
Sample description	No.	GM	Mean	10th	25th	50th	75th	90th	95th
Total	838	0.12	0.22	0.03	0.06	0.11	0.21	0.41	0.64
		(0.10–0.13)	(0.18–0.25)	(0.01–0.05)	(0.05–0.07)	(0.10–0.13)	(0.15–0.27)	(0.33–0.49)	(0.52–0.76)
Race and ethnicity									
Non-Hispanic white	238	0.09 (0.08–0.11)	0.18 (0.12–0.23)	0.03*^a^* (0.02–0.03)	0.05 (0.04–0.06)	0.09 (0.07–0.10)	0.17 (0.14–0.20)	0.31 (0.12–0.51)	0.60 (0.23–0.96)
Non-Hispanic black	161	0.19 (0.14–0.24)	0.32 (0.20–0.43)	0.06*^a^* (0.06–0.07)	0.10 (0.07–0.13)	0.19 (0.11–0.27)	0.33 (0.22–0.44)	0.58 (0.15–1.00)	0.81 (0.44–1.19)
Mexican American	336	0.15 (0.12–0.17)	0.22 (0.18–0.25)	0.05 (0.04–0.06)	0.09 (0.08–0.10)	0.15 (0.12–0.17)	0.27 (0.20–0.34)	0.42 (0.35–0.50)	0.56 (0.40–0.73)
Fish consumption frequency in past 30 days									
0	354	0.08 (0.07–0.10)	0.13 (0.11–0.14)	0.03*^a^* (0.02–0.03)	0.05 (0.04–0.06)	0.08 (0.07–0.09)	0.14 (0.11–0.18)	0.26 (0.21–0.32)	0.38 (0.35–0.40)
1 or 2 times	221	0.14 (0.11–0.16)	0.21 (0.17–0.24)	0.05 (0.04–0.06)	0.07 (0.05–0.09)	0.12 (0.10–0.15)	0.22 (0.14–0.30)	0.39 (0.34–0.44)	0.60 (0.24–0.97)
≥ 3 times	208	0.16 (0.11–0.21)	0.40 (0.24–0.55)	0.04 (0.01–0.06)	0.06 (0.03–0.09)	0.14 (0.09–0.19)	0.30 (0.24–0.36)	0.91 (0.40–1.42)	2.00 (0.39–3.62)
Shellfish consumption frequency in past 30 days									
0	587	0.11 (0.09–0.12)	0.21 (0.17–0.24)	0.03 (0.02–0.04)	0.05 (0.04–0.07)	0.10 (0.08–0.12)	0.18 (0.12–0.24)	0.38 (0.28–0.47)	0.64 (0.55–0.73)
≥ 1 time	195	0.15 (0.11–0.19)	0.27 (0.17–0.36)	0.05 (0.04–0.06)	0.07 (0.02–0.12)	0.14 (0.10–0.18)	0.28 (0.22–0.33)	0.40 (0.26–0.55)	0.66 (0.24–1.08)
Recent hair color or treatment									
No	798	0.12 (0.10–0.13)	0.22 (0.18–0.25)	0.03 (0.01–0.05)	0.06*^a^* (0.05–0.06)	0.11 (0.10–0.13)	0.21 (0.16–0.27)	0.41 (0.31–0.51)	0.66 (0.52–0.79)
Yes	39	0.11 (0.08–0.14)	0.14 (0.12–0.15)	0.04 (–0.00–0.08)*^b^*	0.08 (0.05–0.10)	0.10 (0.07–0.13)	0.13 (0.10–0.16)	0.37 (–0.22–0.97)*^b^*	0.44 (0.35–0.53)

a Bound of CI and percentile are equal because of round-off error.

b Jackknife estimate not stable.

**Table 2:** GM and selected percentile (95% CI) total hair Hg (μg/g): U.S. females 16–49 years of age, NHANES 1999–2000.

				Percentile									
Sample description	No.	GM	Mean	10th	25th	50th	75th	90th	95th
Total	1,726	0.20 (0.16–0.24)	0.47 (0.35–0.58)	0.04 (0.03–0.05)	0.09 (0.07–0.11)	0.19 (0.15–0.23)	0.42 (0.29–0.55)	1.11 (0.54–1.68)	1.73 (1.44–2.02)
Race and ethnicity									
Non-Hispanic white	582	0.20 (0.16–0.24)	0.42 (0.32–0.51)	0.04 (0.02–0.05)	0.09 (0.07–0.11)	0.19 (0.14–0.24)	0.45 (0.21–0.70)	1.17 (0.38–1.96)	1.84 (0.82–2.86)
Non-Hispanic black	356	0.14 (0.11–0.17)	0.48 (0.04–0.91)	0.04 (0.02–0.05)	0.07 (0.05–0.08)	0.13 (0.10–0.16)	0.27 (0.23–0.30)	0.50 (0.35–0.65)	0.88 (0.26–1.50)
Mexican American	593	0.18 (0.15–0.21)	0.28 (0.24–0.31)	0.06 (0.03–0.09)	0.10 (0.07–0.13)	0.18 (0.15–0.20)	0.32 (0.24–0.40)	0.50 (0.46–0.54)	0.78 (0.51–1.05)
Age group (years)	516	0.13*^a^* (0.13–0.20)	0.29 (0.21–0.36)	0.03*^a^* (0.02–0.03)	0.06*^a^* (0.05–0.06)	0.12 (0.09–0.15)	0.27 (0.23–0.31)	0.52 (0.29–0.76)	0.87 (0.51–1.22)
20–29	449	0.18*^a^* (0.18–0.28)	0.39 (0.29–0.48)	0.03 (0.02–0.05)	0.08 (0.07–0.10)	0.18 (0.11–0.24)	0.35 (0.23–0.48)	0.81 (0.65–0.98)	1.43 (1.23–1.64)
30–39	408	0.21 (0.13–0.20)	0.57 (0.33–0.80)	0.04 (0.03–0.05)	0.09 (0.08–0.10)	0.19 (0.12–0.26)	0.40 (0.31–0.48)	1.12 (0.58–1.66)	2.04 (1.55–2.53)
40–49	353	0.26 (0.18–0.28)	0.49 (0.37–0.60)	0.06 (0.04–0.09)	0.11 (0.06–0.16)	0.24 (0.18–0.29)	0.55 (0.40–0.69)	1.39 (1.06–1.72)	1.71 (1.06–2.36)
Fish consumption frequency in past 30 days									
0	639	0.11 (0.08–0.13)	0.25 (0.11–0.38)	0.02 (0.01–0.04)	0.05 (0.03–0.07)	0.10 (0.07–0.14)	0.20 (0.16–0.24)	0.40 (0.25–0.56)	0.55 (0.36–0.74)
1 or 2 times	573	0.20 (0.16–0.25)	0.36 (0.28–0.43)	0.05 (0.04–0.06)	0.10 (0.07–0.13)	0.19 (0.13–0.25)	0.39 (0.33–0.45)	0.79 (0.76–0.81)	1.26 (0.74–1.78)
≥ 3 times	447	0.38 (0.28–0.48)	0.77 (0.59–0.94)	0.09 (0.07–0.10)	0.17 (0.09–0.24)	0.34 (0.24–0.45)	0.81 (0.33–1.30)	1.75 (0.75–2.76)	2.75 (1.99–3.50)
Shellfish consumption frequency in past 30 days									
0	878	0.13 (0.10–0.15)	0.26 (0.20–0.31)	0.03 (0.02–0.04)	0.06 (0.04–0.08)	0.12 (0.10–0.14)	0.25 (0.19–0.32)	0.53 (0.43–0.62)	0.81 (0.24–1.38)
≥ 1 time	782	0.31 (0.25–0.36)	0.64 (0.50–0.77)	0.08 (0.06–0.10)	0.14 (0.12–0.17)	0.28 (0.24–0.32)	0.58 (0.46–0.70)	1.50 (1.29–1.70)	2.22 (1.62–2.83)
Pregnancy status									
Not pregnant	1,429	0.20 (0.16–0.24)	0.47 (0.35–0.58)	0.04 (0.03–0.05)	0.09 (0.07–0.11)	0.19 (0.15–0.23)	0.42 (0.28–0.57)	1.11 (0.50–1.73)	1.71 (1.18–2.25)
Pregnant	292	0.21 (0.15–0.27)	0.43 (0.27–0.58)	0.05 (0.04–0.07)	0.09 (0.08–0.10)	0.17 (0.06–0.28)	0.43 (0.32–0.53)	1.04 (^–^1.09–3.19)*^b^*	1.84 (0.03–3.65)
Recent hair color or treatment									
No	1,089	0.20 (0.16–0.25)	0.43 (0.33–0.52)	0.04 (0.02–0.06)	0.09 (0.07–0.11)	0.19 (0.16–0.23)	0.43 (0.27–0.58)	1.12 (0.57–1.67)	1.70 (0.90–2.49)
Yes	637	0.20 (0.16–0.25)	0.52 (0.34–0.69)	0.04*^a^* (0.04–0.05)	0.09 (0.07–0.11)	0.18 (0.16–0.21)	0.41 (0.32–0.51)	1.10 (^–^0.01–2.21)*^b^*	1.94 (0.74–3.14)
Education (women ≥ 20 years of age)									
< High school	342	0.19 (0.15–0.24)	0.46 (0.28–0.63)	0.05 (0.01–0.08)	0.09 (0.07–0.11)	0.17 (0.13–0.21)	0.34 (0.24–0.43)	0.80 (0.00–1.60)	1.73 (0.95–2.51)
High school	285	0.17 (0.13–0.21)	0.28 (0.22–0.33)	0.04 (0.02–0.06)	0.09 (0.07–0.12)	0.16 (0.11–0.21)	0.31 (0.17–0.44)	0.61 (0.23–0.99)	1.09 (0.60–1.57)
> High school	580	0.24 (0.19–0.30)	0.58 (0.42–0.73)	0.04 (0.03–0.06)	0.10 (0.08–0.12)	0.24 (0.19–0.28)	0.52 (0.32–0.72)	1.41 (0.99–1.82)	2.11 (1.60–2.63)

a Bound of CI and percentile are equal because of round-off error.

b Jackknife estimate not stable.

**Table 3:** Regression model (*R*^2^ = 0.15) and LS means for *y* = log(hair Hg), U.S. children 1–5 years of age, NHANES 1999–2000.

Characteristics	*b* (*p*-Value)	SE	Exp (LS means) (μg/g) (95% CI)
Intercept	−2.64 (< 0.05)	0.09	0.11 (0.10–0.13)
Race/ethnicity			
Non-Hispanic white	—		0.09 (0.08–0.11)
Non-Hispanic black	0.69 (< 0.05)	0.15	0.19 (0.14–0.25)
Mexican American	0.51 (< 0.05)	0.10	0.16 (0.14–0.18)
Fish consumption frequency in past 30 days			
0	—		0.08 (0.07–0.10)
1 or 2 times	0.57 (< 0.05)	0.11	0.14 (0.12–0.17)
≥ 3 times	0.57 (< 0.05)	0.18	0.14 (0.11–0.19)
Shellfish consumption frequency in past 30 days			
0	—		0.11 (0.10–0.13)
≥ 1 time	−0.04 (0.74)	0.13	0.11 (0.08–0.14)
Recent hair color or treatment			
No	—		0.11 (0.10–0.13)
Yes	−0.19 (0.30)	0.18	0.09 (0.06–0.13)

—, Reference level.

**Table 4:** Regression model (*R*^2^ = 0.27) and LS means for *y* = log(hair Hg), U.S. females 16–49 years of age, NHANES 1999–2000.

Characteristics	*b* (*p*-Value)	SE	Exp (LS means) (μg/g) (95% CI)
Intercept	−2.64 ( < 0.05)	0.11	0.19 (0.16–0.22)
Race/ethnicity			
Non-Hispanic white	—		0.20 (0.16–0.24)
Non-Hispanic black	−0.32 (< 0.05)	0.12	0.14 (0.12–0.17)
Mexican American	0.07 (0.44)	0.10	0.21 (0.18–0.25)
Fish consumption frequency in past 30 days			
0	—		0.11 (0.09–0.13)
1 or 2 times	0.55 (< 0.05)	0.10	0.19 (0.6–0.23)
≥3 times	1.05 (< 0.05)	0.14	0.32 (0.24–0.41)
Shellfish consumption frequency in past 30 days			
0	—		0.14 (0.12–0.17)
≥1 time	0.56 (< 0.05)	0.08	0.25 (0.21–0.29)
Recent hair color or treatment			
No	—		0.20 (0.16–0.24)
Yes	−0.11 (0.17)	0.08	0.18 (0.15–0.21)
Pregnancy			
Not pregnant	—		0.19 (0.16–0.22)
Pregnant	0.12 (0.31)	0.12	0.21 (0.17–0.26)
Age groups (years)			
16–19	—		0.15 (0.12–0.19)
20–29	0.15 (0.19)	0.11	0.17 (0.14–0.21)
30–39	0.22 (0.05)	0.11	0.19 (0.15–0.23)
40–49	0.39 (< 0.05)	0.13	0.22 (0.18–0.28)

—, Reference level.
